# Temporal Dynamics Underlying the Modulation of Social Status on Social Attention

**DOI:** 10.1371/journal.pone.0093139

**Published:** 2014-03-25

**Authors:** Mario Dalmaso, Giovanni Galfano, Carol Coricelli, Luigi Castelli

**Affiliations:** Dipartimento di Psicologia dello Sviluppo e della Socializzazione, Università di Padova, Padova, Italy; Birkbeck, University of London, United Kingdom

## Abstract

Fixating someone suddenly moving the eyes is known to trigger a corresponding shift of attention in the observer. This phenomenon, known as gaze-cueing effect, can be modulated as a function of the social status of the individual depicted in the cueing face. Here, in two experiments, we investigated the temporal dynamics underlying this modulation. To this end, a gaze-cueing paradigm was implemented in which centrally-placed faces depicting high- and low-status individuals suddenly shifted the eyes towards a location either spatially congruent or incongruent with that occupied by a subsequent target stimulus. Social status was manipulated by presenting fictive Curriculum Vitae before the experimental phase. In Experiment 1, in which two temporal intervals (50 ms vs. 900 ms) occurred between the direct-gaze face and the averted-gaze face onsets, a stronger gaze-cueing effect in response to high-status faces than low-status faces was observed, irrespective of the time participants were allowed for extracting social information. In Experiment 2, in which two temporal intervals (200 ms vs. 1000 ms) occurred between the averted-gaze face and target onset, a stronger gaze cueing for high-status faces was observed at the shorter interval only. Taken together, these results suggest that information regarding social status is extracted from faces rapidly (Experiment 1), and that the tendency to selectively attend to the locations gazed by high-status individuals may decay with time (Experiment 2).

## Introduction

Social status deeply shapes our social interactions. According to sociologists, social status can be described as “*(…) the prestige accorded to individuals because of the abstract positions they occupy rather than because of immediately observable behavior (…)*” [1]. Generally, high-status individuals tend to use their prestige in order to establish and maintain a set of social norms that define which behavior is permitted, obligated or prohibited within a determined social group [2], leading to hierarchically organized societies [3]. Social status is highly relevant since infancy [4]–[6] and becomes even more important during adolescence [7], [8]. Under an evolutionary perspective, differences in status are also associated to an asymmetric distribution of resources [9], [10]. Therefore, the ability to readily and accurately infer the social status of others represents an essential skill for both humans and nonhuman species to successfully navigate and, in some circumstances, also to survive within social groups characterized by different degrees of complexity [2]. Social status can be inferred from physical traits signalling physical dominance (e.g., facial features, body size, body postures, etc.), especially among nonhuman species such as bees and ants [11], fishes [12], rats [13] and primates [14]. In the case of humans, social status is mainly inferred from specific knowledge about personal characteristics such as educational qualification, job, and material wealth. This implies that, especially in human communities, inferences about social hierarchies are mainly a function of the perceived intellectual capacities and skills of the individuals rather than their perceived physical strength.

Because of the importance of social status in regulating social interactions among humans, several studies have explored the effects of this social variable on human cognitive processes. For instance, it has recently been shown that high-status faces are recognized significantly better than low-status faces, likely because they are coded more accurately [15]. In addition, high-status faces are better attended to and processed more holistically than low-status faces [15]. Furthermore, social status affects the perception of facially-expressed emotions, so that anger is perceived to appear sooner and to last longer on the faces of high-status individuals compared to low-status targets [16]. More relevant for the present study, social status seems to be also involved in regulating social attention. Social attention refers to the ability to shift attention in response to spatial cues provided by others, such as gaze direction, head and body orientation, etc. [17], [18]. This ability allows individuals to discover elements of evolutionary interest in the environment such as dangers, sources of food or potential partners, and to infer, especially in the case of the gaze of others, beliefs, and intentions [19]. Gaze-following abilities have been extensively investigated in humans using a modification of a spatial cueing technique made popular by Posner [20] and known as gaze-cueing paradigm [21]–[31]. This generally consists of presenting a central face with direct gaze that suddenly moves its eyes either rightwards or leftwards. After a variable time interval (Stimulus Onset Asynchrony, SOA) a peripheral target, requiring some kind of response, appears with the same probability in a location either congruent or incongruent with respect to gaze direction. Typically, smaller Reaction Times (RTs) are observed on congruent rather than on incongruent trials, a phenomenon known as gaze-cueing effect that can remain unaltered up to a 1200-ms SOA [26]. After that, the gaze-cueing effect is followed by an inhibitory aftereffect, known as Inhibition of Return (IOR), which consists in observing smaller RTs on incongruent than on congruent trials [32]. However, in order to observe IOR, the SOA has to be much longer than 1200 ms (i.e., 2400 ms). Recently, it has been observed that individuals tend to selectively attend to spatial locations gazed by high- rather than low-status individuals, irrespective of whether information about social status is conveyed through physical traits [33]–[36] or through episodic knowledge [37]. Here, we aimed to clarify the influence of social status as manipulated through episodic learning by focusing on an analysis of temporal dynamics. In the study by Dalmaso et al. [37], participants were first asked to read fictive Curriculum Vitae (CV) conveying information about educational background and job position so that some faces were associated with a high social status and other faces with a low social status. In so doing, unlike other studies [33]–[36] there was no correlation between social status and the perceptual features of the faces used as stimuli. Next, the same facial stimuli were employed in a gaze-cueing task in which each face appeared at fixation with direct gaze for 900 ms, before moving the eyes either rightwards or leftwards. After a fixed 200-ms SOA, a peripheral target appeared in a congruent or incongruent spatial location with respect to gaze direction. Dalmaso et al. [37] reported a reliable gaze-cueing effect in response to high- but not to low-status faces. This is evidence that social status information acquired through episodic learning can shape social attention processes. However, the experiment reported by Dalmaso et al. [37] does not provide any information about the temporal features related to the observed modulation. Two aspects are particularly relevant in this regard.

First, it is unknown whether very fast exposure to a face is sufficient to extract social status information which in turn affects allocation of spatial attention. In Experiment 1, we addressed this issue by keeping the SOA constant at 200 ms, and manipulating the duration of the direct-gaze face frame, that could be either 50 ms or 900 ms. For the long duration, we expected to replicate the results reported by Dalmaso et al. [37]. As for the brief duration, different hypotheses could be put forward. On the one hand, one may hypothesize that retrieving this episodic information may require a substantial amount of time. Because Dalmaso et al. [37] used fixed temporal parameters and left the face with direct gaze available to participants for a considerable time (900 ms), one cannot rule out the possibility that the observed modulation would disappear when shorter exposure times are used. Alternatively, since social status is a critical feature in the regulation of social interaction, one may predict a modulation of social attention processes, as indexed by gaze-cueing, also when faces are presented only briefly. This latter possibility would be supported by evidence showing that the valence associated with person-based representations is automatically retrieved [38].

The second important aspect that had not been addressed by Dalmaso et al. [37] is related to the temporal persistence of the modulation of social attention as a function of social status. In Experiment 2, we addressed this issue by keeping the duration of the direct-gaze face frame constant at 900 ms, and manipulating the duration of SOA, that could be either 200 ms or 1000 ms. In so doing, the former case was a replication of the temporal parameters used by Dalmaso et al. [37]. As for the latter case, on the one hand, one may predict that social status does no longer affect gaze cueing, in that social status information is not relevant for performing the gaze-cueing task, and hence modulations related to differences in social status may disappear. On the other hand, finding a modulation of social attention processes at both the short and the long SOA would cast evidence about the persistent nature of the effects of social status even when this variable is not directly relevant for the task at hand.

## Experiment 1: Materials and Methods

### Participants

Sixty-nine undergraduates (Mean age  = 21.5 years, *SD* = 2.7, 18 males) took part in the experiment on a voluntary basis. All were naive as to the purpose of the study and reported normal or corrected- to-normal vision.

### Ethics statement

All participants provided a written informed consent prior to taking part in the experiment. The Ethics Committee for Psychological Research at the University of Padova approved the study.

### Apparatus, stimuli, and procedure

Six full-colour photos of older male adults aged between 50 and 60 years bearing a neutral expression were extracted from “The Color FERET Database” [39] (00474_940519_fa, 00714_941201_fa, 00739_941201_fb, 00919_960620_fa, 00950_960627_fa, 00955_960627_fb; see also [37]). Any element of asymmetry (e.g., moles, birthmark, etc.) was removed using The Gimp 2.6 (The Gimp Team, http://www.gimp.org).

For each face there were three different versions: one with direct gaze (i.e., the original photograph), one with gaze averted rightwards and one with gaze averted leftwards. The averted-gaze photographs were obtained by moving the irises 0.25° to the right or to the left from the original central position using The Gimp 2.6. Participants sat approximately 57 cm away from a 17-inch monitor (1024×768 pixel, 60 Hz). A PC running E-Prime 1.1 handled timing and stimuli presentation. A standard keyboard collected manual responses.

The whole experiment was composed of three computer-based phases: a learning phase, in which participants were asked to learn the social status of the face stimuli; an experimental phase, in which the same faces were employed in the gaze-cueing task; a manipulation check, aimed to verify whether participants remembered the association between each face and the corresponding social status studied during the learning phase. In all phases, each face was presented alone, with constant size (21.2°×14°), in a central position and against a black background.

The learning phase consisted of presenting each face singularly, accompanied with a fictive CV that appeared in white letters (18-point Courier New) above the face. Three faces were paired to a high-status profile (1^st^ CV: ‘Dean of a Faculty of Architecture. President of the European Eco-Sustainable Constructions Society’; 2^nd^ CV: ‘Dean of a Faculty of Economy. He is director of the journal “Economy & Management”; 3^rd^ CV: ‘Dean of a Faculty of Medicine. He developed an innovative surgical techniques for the treatment of digestive tract tumours’), whereas the other three faces were paired to a low-status profile (1^st^ CV: ‘Retired factory worker. He did not complete primary school’; 2^nd^ CV: ‘Retired agricultural worker. After the elementary school he worked as a labourer in a farm’; 3^rd^ CV: Retired factory worker. After the elementary school he worked in the textile industry'). The association between faces and profiles was randomly determined for each participant. In so doing, we minimized the eventual influence of the physiognomic traits of the stimulus faces. Status was mainly related to educational/academic information that was highly relevant for the participants recruited in the study (i.e., undergraduate students; see also [37]). Participants were asked to memorize each face identity and the corresponding CV, with no time limits. To move from a face to another one, participants were asked to press the spacebar. When participants had visualized all the 6 faces, a categorization task was administered in order to verify learning. This task consisted of presenting each face for 900 ms without CV. Within that time, participants were required to categorize each face as depicting a high- or a low-status individual by pressing the ‘Y’ and the ‘B’ keys, respectively. Each face appeared twice for a total of 12 trials. The green text ‘CORRECT’ or the red texts ‘ERROR’ or ‘FASTER’ appeared centrally for 2000 ms in case of a correct, an incorrect or a missing response, respectively. In case participants committed at least one error in these 12 trials, the categorization task was administered again. Moreover, in case participants were unable to complete successfully the categorization task after 8 cycles, they were presented again with both faces and their associated CVs.

After the learning phase was successfully completed, the experimental phase started. This consisted of a gaze-cueing task in which the same faces used in the learning phase were employed. Each trial began with the presentation of a white fixation cross (0.82°) in the centre of the screen for 900 ms, followed by a central face with direct gaze. After either 50 ms or 900 ms, the same face appeared with the gaze averted either rightwards or leftwards. After a fixed SOA of 200 ms, a white target letter (‘L’ or ‘T’, 0.82°) appeared 11° rightwards or leftwards from the centre of the screen with the same probability. The averted-gaze face and the target letter remained visible until a response was provided or 1500 ms had elapsed, whichever came first. Participants were informed that gaze direction was uninformative with regard to the target location, they were instructed to maintain fixation at the centre of the screen, to ignore gaze direction, and to respond as fast and accurately as possible. Half of the participants responded by pressing the ‘K’ key with their right index finger in case the target was a ‘L’, and the ‘D’ key with their left finger in case the target was a ‘T’. The remaining participants responded using the opposite mapping. In the case of a wrong or a missing response, the central red text ‘ERROR’ or ‘NO RESPONSE’ appeared on the screen for 1500 ms. There was a practice block composed by 10 trials followed by 3 experimental blocks each composed of 96 trials, for a total of 288 experimental trials presented in a random order.

After the experimental phase, participants were asked to take part in the manipulation check task. This was identical to the categorization task of the learning phase, the only exceptions being that a single cycle was presented and that there was no time limit for responding. This latter change had the purpose of maximizing accuracy in the responses. At the end of the experiment the participants were thanked and debriefed. The whole procedure took about 1 hour.

## Experiment 1: Results and Discussion

Participants who committed at least one error during the manipulation check (*N* = 13) and with a percentage of errors during the experimental phase that fell 2 SD above the mean (*N* = 1) were excluded from the analyses, leaving 55 participants for the analyses (Mean age  = 21.7 years, *SD* = 2.8, 13 males). Then, incorrect responses were removed and analysed separately (2.68% of total trials).

**Figure 1:**
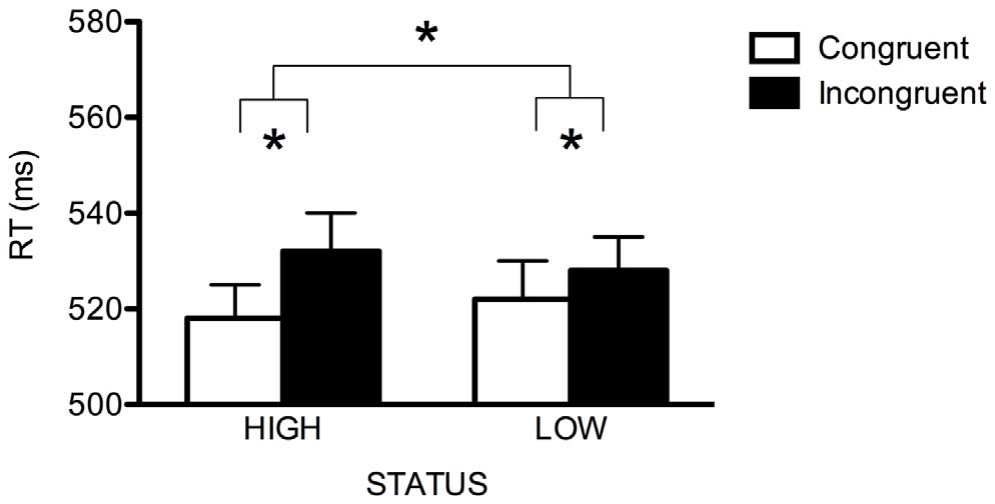
Median RT (ms) as a function of Cue-Target spatial congruency and Status in Experiment 1. Error bars represent SEM. Asterisk denotes *p*<.05.

A repeated-measures ANOVA was performed on median RTs with Cue-target spatial congruency (2: congruent vs. incongruent), Direct-gaze face duration (2: 50 ms vs. 900 ms) and Status (2: high vs. low) as within-participant factors. We used medians because they reduce the effect of outliers. The main effect of Cue-target spatial congruency was significant, *F*(1,54) = 37.120, *p*<. 001, η^2^ *_p_* = .407, owing to smaller RTs on congruent (*M* = 520 ms, *SE* = 7.4) than on incongruent (*M* = 530 ms, *SE* = 7.3) trials, as well as the Cue-target spatial congruency × Status interaction, *F*(1,54) = 6.388, *p* = .014, η^2^ *_p_* = .106. Paired comparisons between congruent and incongruent trials divided by status revealed that participants oriented their attention in response to the averted gaze of both high, *t*(54) = 6.440, *p*<.001, and low, *t*(54) = 2.527, *p* = .014, status faces, but the effect was greater in the former case (15 ms vs. 6 ms; see Figure 1). Critically, the three-way Cue-target spatial congruency × Direct-gaze face duration × Status interaction was not significant (*F*<1, *p* = .829), confirming a comparable effect of social status on gaze-cueing irrespective of direct-gaze face duration (see Table 1). No other significant main effects or interactions emerged (all *F*s<1.5, *p*s>.23). In order to strengthen our conclusions, we conducted two separate ANOVAs for each of the two levels assumed by the Direct-gaze face duration (i.e., 50 ms vs. 900 ms). At the 50-ms duration, the main effect of Cue-target spatial congruency was significant, *F*(1,54) = 17.512, *p*<. 001, η^2^ *_p_* = .245, owing to smaller RT on congruent (*M* = 522 ms, *SE* = 7.5) than on incongruent (*M* = 531 ms, *SE* = 7.4) trials, while the Cue-target spatial congruency × Status approached statistical significance, *F*(1,54) = 3.869, *p* = . 054, η^2^ *_p_* = .067. However, paired comparisons between congruent and incongruent trials divided by status confirmed that participants oriented their attention in response to the averted gaze of high, *t*(54) = 5.024, *p*<.001, but not low, *t*(54) = 1.642, *p* = .106, status faces. Furthermore, in the attempt to obtain addition evidence about the presence of gaze cueing at the 50-ms SOA only in response to high-status faces, data were also submitted to Bayesian analyses which allows one to disentangle which model (null vs. alternative hypothesis) is more strongly supported by the available data. The Bayesian Information Criterion (BIC) was computed following the procedure put forward by Masson [40]. This analysis showed that the posterior probability favouring the hypothesis that gaze cueing was present in response to high-status faces was *p*_BIC_(H1 | D)>0.99. In contrast, the posterior probability favouring the hypothesis that gaze cueing was present in response to low-status faces was *p*_BIC_(H1 | D) = 0.34. According to the conventional categorization of degrees of evidence [40], the obtained posterior probabilities for the alternative hypothesis constitute a “very strong” evidence for the conclusion that a gaze-cueing effect is present in response to high-status faces, whereas no cueing effect is present in response to low-status faces. At the 900-ms duration, the main effect of Cue-target spatial congruency was significant, *F*(1,54) = 24.744, *p*<. 001, η^2^ *_p_* = .314, owing to smaller RT on congruent (*M* = 517 ms, *SE* = 7.6) than on incongruent (*M* = 529 ms, *SE* = 7.6) trials, as well as the Cue-target spatial congruency × Status interaction, *F*(1,54) = 4.528, *p* = . 038, η^2^ *_p_* = .077. Paired comparisons between congruent and incongruent trials divided by status revealed that participants oriented their attention in response to the averted gaze of both high, *t*(54) = 4.6, *p*<.001, and low, *t*(54) = 2.625, *p* = .011, status faces, but the effect was greater in the former case (16 ms vs. 7 ms).

**Table 1:** Median RT (ms) and percentage of errors (%) for each condition in Experiment 1 and 2.

	EXPERIMENT 1								EXPERIMENT 2							
	Direct-gaze face duration 50-ms				Direct-gaze face duration 900-ms				Averted-gaze face duration 200-ms SOA				Averted-gaze face duration 1000-ms SOA			
	High Status		Low Status		High Status		Low Status		High Status		Low Status		High Status		Low Status	
	C	I	C	I	C	I	C	I	C	I	C	I	C	I	C	I
RT	520 (7)	534 (8)	523 (8)	528 (7)	515 (8)	531 (8)	520 (8)	527 (8)	543 (10)	550 (10)	546 (10)	550 (9)	523 (9)	535 (10)	518 (9)	542 (10)
% E	2.4 (.38)	1.7 (.29)	1.9 (.34)	2 (.27)	2.1 (.36)	2.4 (.48)	3 (.4)	2.3 (.37)	2.3 (.4)	2.8 (.4)	1.9 (.37)	2.5 (.41)	1.9 (.3)	2.4 (.47)	1.1 (.24)	1.9 (.39)

Values in brackets are SEM. C =  congruent trials; I =  incongruent trials.

A second repeated-measures ANOVA was conducted on the percentage of errors with Cue-target spatial congruency (2: congruent vs. incongruent), Direct-gaze face duration (2: 50 ms vs. 900 ms) and Status (2: high vs. low) as within-participant factors. The main effect of Direct-gaze face duration approached significance, *F*(1,54) = 3.472, *p* = .068, η^2^ *_p_* = .06, reflecting the tendency to commit more errors at the longer (*M* = 2.4%, *SE* = .25) than at the shorter (*M* = 2%, *SE* = .17) duration. Moreover, also the Cue-target spatial congruency × Direct-gaze face duration × Status interaction approached significance, *F*(1,54) = 3.88, *p* = .054, η^2^ *_p_* = .067. However, the critical paired comparisons between congruent and incongruent trials divided by duration and status revealed no differences among the critical conditions (*p*s>.13; see Table 1). No other significant main effects or interactions emerged (all *F*s<1.33, *p*s>.253). Thus, the data were unlikely to be affected by any speed–accuracy trade-off, a pattern of results in line with previous literature. Indeed, while other spatial cueing paradigms, like those in which symbolic endogenous cues are used [41], can consistently affect both RTs and accuracy, in the case of gaze-cueing paradigms attentional modulations are mainly reflected on RTs [24], [34], [37].

Although not strictly relevant to our hypotheses, we performed additional analyses on RTs and Accuracy including the between-participants factor of gender. In fact, some evidence reported an influence of gender on the modulation of social status on attentional processes [42]. The only significant result involving gender was the main effect of this factor observed for RTs, *F*(1,53) = 4.729, *p* = . 034, η^2^ *_p_* = .082, with males who overall showed smaller RTs (*M* = 497 ms, *SE* = 14.6) with respect to females (*M* = 533 ms, *SE* = 8.1). However, these results should be taken with prudence due to the unbalanced number of females (*N* = 42) and males (*N* = 13).

The results from this experiment are interesting mainly for three reasons. First, they are in line with those reported by Dalmaso et al. [37], namely that the tendency to attend to the spatial location indicated by other's gaze direction is more pronounced in response to high- rather than to low-status faces. Second, in Dalmaso et al. [37], faces from different age levels were used and, in each experimental condition, status covaried with age to help building episodic knowledge. In contrast, here we used faces from a single age level (older adults). Hence, the present findings cast stronger evidence that gaze cueing is influenced by social status, in that participants could not rely upon any categorical cue to retrieve episodic knowledge about status. Finally, and more importantly, the observed gaze cueing modulation argues in favour of a rapid integration of social status and gaze cues. This would confirm that individuals are particularly sensitive to signals of social status and process them efficiently.

## Experiment 2: Materials and Methods

### Participants

Seventy-six undergraduates (Mean age  = 23.8 years, *SD* = 4.9, 26 males) took part in the experiment on a voluntary basis. None of them had taken part in the previous experiment. All were naive as to the purpose of the study and reported normal or corrected-to-normal vision.

### Ethics statement

All participants provided a written informed consent prior to taking part in the experiment. The Ethics Committee for Psychological Research at the University of Padova approved the study.

### Apparatus, stimuli, and procedure

Apparatus, stimuli and procedure were the same as in Experiment 1, with the following exceptions: the duration of the direct-gaze face frame was held constant at 900 ms, as in Dalmaso et al. [37], and two SOAs of 200 ms and 1000 ms were used.

## Experiment 2: Results and Discussion

We used the same data reduction rationale as in Experiment 1. Participants who committed at least 1 error during the manipulation check (*N* = 19) and with a percentage of errors during the experimental phase that fell 2 SD above the mean (*N* = 4) were excluded from the analyses, leaving 53 participants for the analyses (Mean age  = 23.4 years, *SD* = 2.7, 19 males). Then, incorrect responses were removed and analysed separately (2.09% of the total trials).

A repeated-measures ANOVA was performed on median RTs with Cue-target spatial congruency (2: congruent vs. incongruent), SOA (2: 200 ms vs. 1000 ms) and Status (2: high vs. low) as within-participants factors. The main effect of Cue-target spatial congruency was significant, *F*(1,52) = 24.777, *p*<.001, η^2^ *_p_* = .323, owing to smaller RTs on congruent (*M* = 533 ms, *SE* = 9.1) than on incongruent trials (*M* = 544 ms, *SE* = 9.5), as well as the main effect of SOA, *F*(1,52) = 35.192, *p*<.001, η^2^ *_p_* = .404, owing to smaller RT at the longer (*M* = 529 ms, *SE* = 9.3) than at the shorter (*M* = 547 ms, *SE* = 9.4) SOA. The Cue-target spatial congruency × SOA interaction was also significant, *F*(1,52) = 8.343, *p* = .006, η^2^ *_p_* = .138. Paired comparisons between congruent and incongruent trials divided by SOA revealed that the cueing effect was significant both at the shorter, *t*(52) = 2.327, *p* = .024, and at the longer, *t*(52) = 4.669, *p*<.001, SOA, but the effect was larger in the latter case (5 ms vs. 18 ms). The two-way Cue-target spatial congruency × Status interaction did not yield a significant effect (*F* = 1.61, *p* = .209), whereas the three-way Cue-target spatial congruency × SOA × Status interaction was statistically significant, *F*(1,52) = 4.551, *p* = .038, η^2^ *_p_* = .080. Paired comparisons between congruent and incongruent trials divided by SOA and status revealed that the cueing effect was significant for high-status faces both at the shorter and at the longer SOA, in both cases *t*(52) = 2.531, *p* = .014, and also for the low-status faces but only at the longer SOA, *t*(52) = 5.72, *p*<.001. At the shorter SOA, the cueing effect for low status faces was not significant, *t*(52) = .921, *p* = .361 (see Table 1). No other significant main effects or interactions emerged (all *F*s<1, *p*s>.331).

A second repeated-measures ANOVA was conducted on the percentage of errors with the same factors as above. The main effect of Cue-target spatial congruency was significant, *F*(1,52) = 7.083, *p* = .010, η^2^ *_p_* = .12, owing to more errors on incongruent (*M* = 2.4%, *SE* = .26) than on congruent (*M* = 1.8%, *SE* = .2) trials. The main effect of SOA approached significance, *F*(1,52) = 3.943, *p* = .052, η^2^ *_p_* = .07, reflecting more errors at the shorter (*M* = 2.4%, *SE* = .26) than at the longer (*M* = 1.8%, *SE* = .24) SOA, as well the main effect of Status, *F*(1,52) = 3.487, *p* = .067, η^2^ *_p_* = .063, reflecting more errors in response to high- (*M* = 2.3%, *SE* = .25) than to low-status (*M* = 1.8%, *SE* = .23) faces. No other significant main effects or interactions emerged (all *F*s<1, *p*s>.538). Thus, no speed–accuracy trade-off affected the data.

As for Experiment 1, we performed additional analyses on RTs and Accuracy including the between-participants factor of gender. The only significant result involving this factor was the Cue-target spatial congruency × Gender, *F*(1,51) = 4.284, *p* = .044, η^2^ *_p_* = .077, observed for RTs. Paired comparisons between congruent and incongruent trials divided by gender revealed that the cueing effect was significant among females, *t*(33) = 5.395, *p*<.001, but not among males, *t*(18) = 1.394, *p* = .18. This result is consistent with previous studies in which males, compared to female participants, showed a reduced gaze-cueing effect [21]. However, as for Experiment 1, these results should be taken with prudence due to the unbalanced number of females (*N* = 34) and males (*N* = 19).

In line with Experiment 1 and with Dalmaso et al. [37], in Experiment 2 a reliable gaze-cueing effect emerged in response to the averted gaze of high- but not low-status individuals at the shorter SOA, whereas at the longer SOA a reliable gaze-cueing effect emerged irrespective of the social status of the faces. This pattern of results suggests that the tendency to selectively attend to the locations gazed by high-status individuals decays with time.

## Conclusions

Despite the crucial role that social status plays in regulating interactions among humans, only in recent years researchers started to systematically investigate the effect of social hierarchies on our cognitive mechanisms [43], including perceptual [16], [44] and memory [15] processes. More relevant for the present study, social status seems to be also involved in regulating social attention [37]. As for the temporal dynamics underlying the modulation of attentional processes elicited by social information conveyed by faces, there is evidence of significant effects even at very brief exposures, such as 50 ms or even less [45]–[47]. However, social information in all these cases was delivered through physical cues such as emotional expressions and ethnic membership. The present set of experiments was designed to clarify the temporal aspects of the interplay between social factors and attentional processes when social information is conveyed through episodic learning rather than physical cues.

To this end, two experiments were conducted manipulating the critical temporal intervals related to a standard gaze cueing paradigm. In Experiment 1, we varied the temporal duration of the direct-gaze face, which could be 50 ms or 900 ms. A reliable gaze-cueing effect in response both to high- and low-status faces was observed, but the effect was greater in the former case. Critically, this modulation was not affected by the temporal duration of the manipulated interval, a result that argues in favour of a rapid integration of social status and gaze cues. It is worth noting that, unlike previous studies in which the manipulation was based on changes in the physical features of the faces used as stimuli, here we observed a modulation due to non-visual information associated with faces. However, while on the one hand it is highly unlikely that the current results have been affected by some physical properties of our facial stimuli (e.g., attractiveness, dominance, trustworthiness, etc.), as the assignment of the status was random, on the other hand we do not have any concurrent measurement to properly support this argument. We know from previous literature that such features can modulate gaze cueing [33]–[35] and therefore in future studies it will be important to also assess them through questionnaires and self-reported measures. Nevertheless, the overall findings suggest the possibility that a top-down process based on previous knowledge stored in memory can also readily impact onto our social attention mechanism, at least in the case of social status.

In Experiment 2, the crucial temporal manipulation concerned SOA duration, which could be either 200 ms or 1000 ms. At the 200-ms SOA, a reliable gaze-cueing effect emerged in response to high- but not low-status faces whereas, at the 1000-ms SOA, a reliable gaze-cueing effect emerged irrespectively of the status of the face. This pattern suggests that the tendency to selectively attend to spatial locations gazed by high-status individuals decays with time. This result is in line with previous evidence suggesting that social status, conveyed through physical traits, produces only short-term effects on social attention [33]. Therefore, it seems that social status can mainly impact the reflexive components of the gaze-cueing effect, which are typically more apparent at shorter SOAs [23], [24]. Taken together, the results of Experiment 1 and 2 provide converging evidence that high-status individuals are rapidly accorded a stronger attentional priority - as indexed by the effectiveness of their gaze in pushing the observer's attention - but also that status-related differences induce a short-lived influence upon attentional processes. Indeed, gaze cueing is affected by differences in status only at the short SOA.

The interplay between status and social attention may be adaptive and functional for the regulation of group processes. Interestingly, the first study reporting a modulation of social status on social attention has been conducted on non-human primates [48]. More specifically, Shepherd et al. [48] reported that high-status monkeys oriented their attention only in response to gaze direction of same-status peers, whereas low-status monkeys oriented their attention in response to both high- and low-status faces. The phylogenetic relevance of social status emerging from this study strengthens the idea that the ability to readily detect and respond to signals of social status is a key factor to successfully navigate within social groups. Moreover, the fact that, even in non-humans primates, social attention can be affected by hierarchical differences, suggests the possible existence of a cognitive mechanism, shared with other animal species, devoted to the elaboration and monitoring of high-status individuals [49], [50]. Supportive evidence in favour of this hypothesis comes also from studies using neuroimaging techniques. Recent research provided the first evidence that some neural circuits can be modulated by social status, both in humans [51]–[56] and in nonhuman primates [57]. These pioneering results strengthen the notion that individuals from several species are equipped with a neural network devoted to the elaboration of social status information.

In sum, our findings show that social status information can rapidly be extracted from faces on the basis of previous episodic learning and that differences in gaze cueing as a function of social status disappear with time. Future studies may provide further insight about the temporal dynamics underlying the modulation of social attention as a function of social status by combining behavioural and high-temporal resolution electrophysiological measures. This may also help understanding the neural bases of the interplay between social status and social attention processes.
